# Serotonergic Neurons in the Brain and Gnathal Ganglion of Larval *Spodoptera frugiperda*

**DOI:** 10.3389/fnana.2022.844171

**Published:** 2022-03-10

**Authors:** Jia-Jia Zhang, Long-Long Sun, Ya-Nan Wang, Gui-Ying Xie, Shi-Heng An, Wen-Bo Chen, Qing-Bo Tang, Xin-Cheng Zhao

**Affiliations:** Henan International Joint Laboratory of Green Pest Control, College of Plant Protection, Henan Agricultural University, Zhengzhou, China

**Keywords:** brain, gnathal ganglion, immunoreactivity, neuropils, serotonin, *Spodoptera frugiperda*, taste

## Abstract

The fall armyworm *Spodoptera frugiperda* (*S. frugiperda*) (Lepidoptera: Noctuidae) is a worldwide, disruptive, agricultural pest species. The larvae of *S. frugiperda* feed on seedling, leave, and kernel of crops with chewing mouthparts, resulting in reduced crop yields. Serotonin is an important biogenic amine acting as a neural circuit modulator known to mediate lots of behaviors including feeding in insects. In order to explore the serotonergic neural network in the nervous system of larval *S. frugiperda*, we performed immunohistochemical experiments to examine the neuropil structure of the brain and the gnathal ganglion with antisynapsin and to examine their serotonergic neurons with antiserotonin serum. Our data show that the brain of larval *S. frugiperda* contains three neuromeres: the tritocerebrum, the deutocerebrum, and the protocerebrum. The gnathal ganglion also contains three neuromeres: the mandibular neuromere, the maxillary neuromere, and the labial neuromere. There are about 40 serotonergic neurons in the brain and about 24 serotonergic neurons in the gnathal ganglion. Most of these neurons are wide-field neurons giving off processes in several neuropils of the brain and the gnathal ganglion. Serotonergic neuron processes are mainly present in the protocerebrum. A pair of serotonergic neurons associated with the deutocerebrum has arborizations in the contralateral antennal lobe and bilateral superior lateral protocerebra. In the gnathal ganglion, the serotonergic neuron processes are also widespread throughout the neuropil and some process projections extend to the tritocerebrum. These findings on the serotonergic neuron network in larval *S. frugiperda* allow us to explore the important roles of serotonin in feeding and find a potential approach to modulate the feeding behavior of the gluttonous pest and reduce its damage.

## Introduction

The fall armyworm (FAW) *Spodoptera frugiperda* (*S. frugiperda*) (Lepidoptera: Noctuidae) is a disruptive agricultural pest species and shows a high potential to cause crop yield loss due to its polyphagy, gluttony, high mobility, and high reproductivity. FAW can feed on more than 80 crops, including maize, wheat, sorghum, millet, sugarcane, vegetable crops, and cotton (Montezano et al., 2018). Larvae of FAW can feed on seedlings, leaves, developing tassel, and kernel with chewing mouthparts, which could reduce the photosynthetic area, grain quality, and ultimately reduce the yield. FAW is native to the Americas, but has now spread globally. In China, FAW was first found in southeast of Yunnan Province in 2019 and rapidly spreads through eastern China (Wu et al., 2019, 2021). Once this pest species is established in a country or area, it may not be possible to eradicate it because of its high adaptation. It is necessary to develop the strategies for the sustainable control of *S. frugiperda* by exploring any potential target at the levels of molecule, physiology, and behavior.

Serotonin [5-hydroxytryptamine (5-HT)] is a biogenic amine, acting as neurotransimitter, neuromodulator, or neurohormone in a wide range of organisms, including insects (Nässel, 1988; Vleugels et al., 2015). The immunohistochemical experiments with serotonin antiserum showed that serotonergic neurons of insect species are limited in number, but the project processes were widely distributed in both the peripheral and the central nervous system (Klemm et al., 1984; Lange et al., 1988; Nässel, 1988; Homberg and Hildebrand, 1989a,b; Breidbach, 1990; Boleli and Paulino-Simões, 1999; Leitinger et al., 1999; Settembrini and Villar, 2004; Dacks et al., 2006; Liu et al., 2011; Huser et al., 2012; van der Woude and Smid, 2017; Tang et al., 2019; Tierney, 2020). The widespread serotonergic neurons are involved in multiple effects in a variety of behaviors and physiological activities, including vision, olfaction, audition, feeding, flight, aggregation, aggression, sleep, learning and memory, circadian rhythms, immunity, stress, metabolism, growth, and reproduction (Kloppenburg and Mercer, 2008; Anstey et al., 2009; Qi et al., 2014, 2016; Tang et al., 2019; Tierney, 2020).

Particularly, the modulation of serotonin on feeding-related processes has been intensively studied in several insect species, including locust, aphid (Kaufmann et al., 2004), bugs (Orchard, 2006), bees (French et al., 2014), mosquitoes (Novak and Rowley, 1994), and flies (Haselton et al., 2009; Albin et al., 2015; Schoofs et al., 2018; Lyu et al., 2021). The serotonergic modulation is involved in feeding states, e.g., hunger and satiety and in a sequence of discrete events of feeding, such as, food detection, salivary secretion, food intake, and ingestion of food (Tierney, 2020). Elevated serotonin inhibited the proboscis extension, decreased feeding time, and reduced sucrose consumption in cockroach, honeybees, ants, mosquitoes, blow fly, and fresh fly (Cohen, 2001; Dacks et al., 2003; Haselton et al., 2009; Falibene et al., 2012; French et al., 2014; Kinney et al., 2014). Injected serotonin showed synergistic suppression of pymetrozine, an insecticide for aphid and locust (Kaufmann et al., 2004). These reports supported the association between serotonin and satiety. In some experiments, however, serotonin was also found to be associated with hunger. Activation of a subset of serotonergic neurons in the brain of *Drosophila* could increase food intake (Albin et al., 2015). Depletion of serotonin in bug *Rhodnius prolixus* and mosquito *Aedes triseriatus* suppressed blood intake (Cook and Orchard, 1990; Novak and Rowley, 1994).

Recently, measurements of high-performance liquid chromatography showed that serotonin is present in the brain and the digestive tract of larval *S. frugiperda* (Oyarzabal-Armendariz et al., 2021). After fed with azadirachtin, the amount of serotonin increased in the larval *S. frugiperda* brain, but decreased in the intestine. The increased serotonin could alter activities in memory, learning, sleep, and locomotor, while the decreased serotonin reduce the peristalsis movements (Oyarzabal-Armendariz et al., 2021). Therefore, the antifeedant and repellent effects of azadirachtin on *S. frugiperda* might be mediated by serotonin signal (Lin et al., 2021; Oyarzabal-Armendariz et al., 2021). Here, we performed immunohistochemistry with antiserotonin serum to examine the distribution of serotonergic neurons in the central nervous system of larval *S. frugiperda*. We provide the first comprehensive description of the serotonergic neuronal network in *S. frugiperda* larvae, which is essential for understanding the neural mechanism of feeding-related modulation.

## Materials and Methods

### Insects

Larval *S. frugiperda* was reared on an artificial diet (wheat bran 40 g, yeast powder 34 g, casein 25 g, sorbic acid 2 g, corn meal 100 g, vitamin composite powders 4 g, methylparaben 4 g, agar 18 g, and distilled water 900 ml) in the laboratory under 16/8 light/dark, at 27 ± 1°C and 75% relative humidity. Larvae at the second day of 5th instar were used for the immunohistochemistry experiments. Adults were fed on a 10% sucrose solution.

### Immunohistochemistry for Synapsin and Serotonin

In order to examine the distribution of serotonin-immunoreactive neurons in the brain and the gnathal ganglion, immunohistochemistry with the antisynapsin for labeling the neuropil structure and antiserotonin for labeling the serotonergic neurons was performed. The detailed procedures were described in previous studies (Zhao et al., 2016; Tang et al., 2019). The preparations were dissected. The brain and the gnathal ganglion were isolated from the insect body in Ringer’s solution (150 mM NaCl, 3 mM CaCl_2_, 3 mM KCl, 25 mM sucrose, and 10 mM N-tris(hydroxymethyl)methyl-2-aminoethanesulfonic acid, pH 6.9) and fixed in 4% paraformaldehyde (PFA) solution (4% PFA in 0.1 M phosphate buffer) for 2–4 h at room temperature. Followed the rinse with phosphate-buffered saline (PBS) (684 mM NaCl, 13 mM KCl, 50.7 mM Na_2_HPO_4_, and 5 mM KH_2_PO_4_, pH 7.4) for 6 times, each time 10 min, the brain and the gnathal ganglion were preincubated in 10% normal goat serum (NGS) (Sigma Aldrich, St Louis, Mosby, United States) in PBS containing 0.5% Triton X-100 (PBST) for 3 h at room temperature to minimize the non-specific staining. Next, the brain and the gnathal ganglion were incubated in the primary antibodies, anti-SYNORF1 (1:100, Developmental Studies Hybridoma Bank, University of Iowa, Iowa City, Iowa, United States) and antiserotonin serum (1:4,000, Immunostar Incorporation, Hudson, Wisconsin, United States), in PBST containing 5% NGS for 5 days at 4°C. After that, the samples were rinsed with PBS for 6 × 20 min and then incubated in the secondary antibodies, Alexa Fluor 488 conjugated goat antimouse (1:400, Invitrogen, Eugene, Oregon, United States) and Alexa Fluor 633 conjugated goat antirabbit (1:400, Invitrogen, Eugene, Oregon, United States) in PBST containing 5% NGS for 3 days at 4°C. Finally, the samples were rinsed 6 × 20 min with PBS, dehydrated in a series ethanol (50, 70, 90, and 96% and 2 × 100%, 10 min each time), cleared in methyl salicylate, and mounted in Permount.

### Image Data Acquisition and Analysis

All the images were obtained by laser scanning confocal microscope (Nikon A1, Japan) with 10X/2.2 air objective. Fluorescent dyes of Alexa Fluor 488 and Alexa Fluor 633 were excited by a 488-nm Argon laser and a 633-nm HeNe laser, respectively. The resolution of image is 1,024 × 1,024 and the interval is 2–3 μm. The value of high voltage (HV) is set at 35–50 and the laser intensity and other parameters are adjusted during scanning.

Confocal image data format was converted into tag image file format by Image J software [version 1.53f51, National Institutes of Health (NIH), United States]. Software of Amira version 5.3 (Visage Imaging, Fürth, Germany) was then used to analyze the image stacks. Neuropil structures and nerves were reconstructed by using the tool of LabelField of Amira and serotonin-immunoreactive neurons were reconstructed by using the module of SkeletonTree. Volumes of neuropils and cell bodies were measured by using the tool of TissueStatistics. Adobe Photoshop was used to adjust color, brightness, and contrast of confocal image when necessary and the image panels were edited by Adobe Illustrator 2021 (Adobe System, San Jose, California, United States). ANOVA and the bar chart of the average diameter and volume of cell bodies were performed by using GraphPad Prism version 9.0 (GraphPad Software Incorporation, San Diego, California, United States). The nomenclature of neuroanatomical structures, serotonin-immunoreactive neurons, and abbreviations suggested by Ito et al. (2014) and Tang et al. (2019) were used for larval *S. frugiperda*.

## Results

In total, we performed immunohistochemical staining with anti-SYNORF1 and antiserotonin serum on 30 preparations, of which 14 preparations were stained successfully, 10 preparations were stained weakly, and six preparations were unstained. Four brains and five gnathal ganglia were used for examining the neuroanatomical structures and the distribution of serotonergic neurons.

### Anatomy of Larval *Spodoptera frugiperda* Brain

**FIGURE 1:**
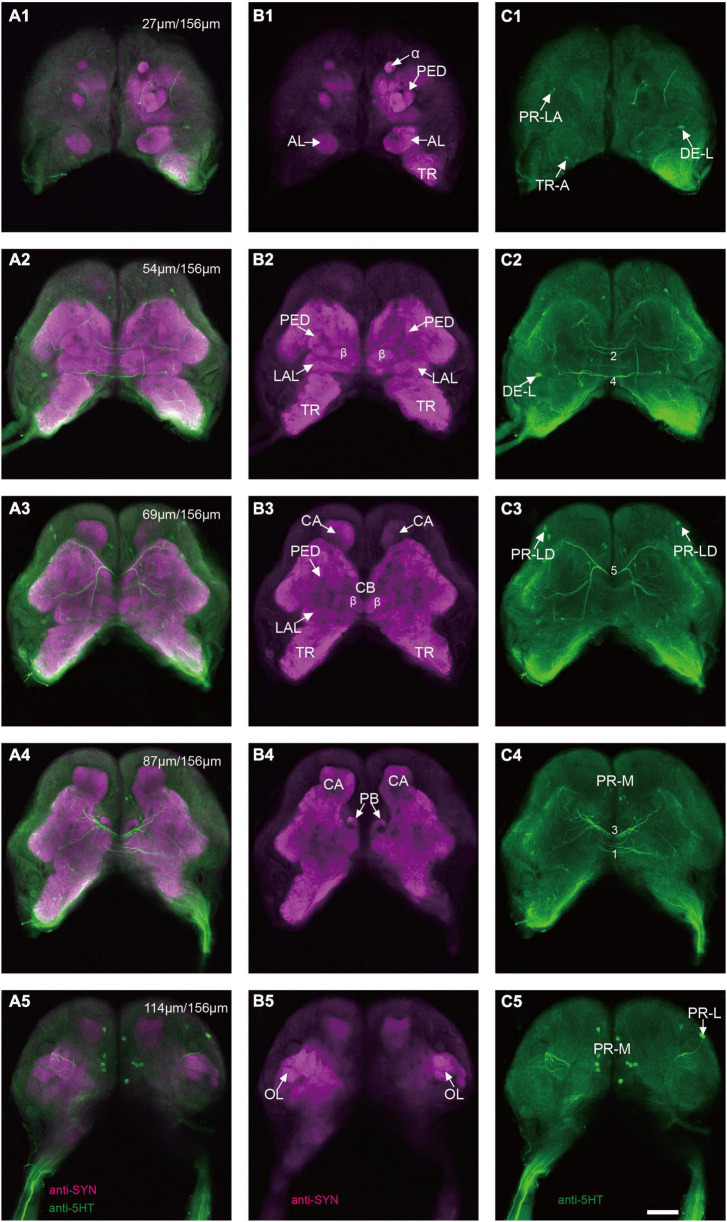
Confocal images of the brain of *Spodoptera frugiperda* larvae. **(A1–A5)** Merged confocal image showing the neuropil (magenta) and the serotonin-immunoreactive neurons (green). **(B1–B5)** Confocal image showing the neuropils of the brain. **(C1–C5)** Confocal image showing the serotonin-immunoreactive neurons in the brain. α, alpha lobe; AL, antennal lobe; CA, calyx; CB, central body; LAL, lateral accessory lobe; OL, optic lobe; PB,: protocerebral bridge; PED, pedunculus; TR, tritocerebrum. Scale bars, 100 μm.

**FIGURE 2:**
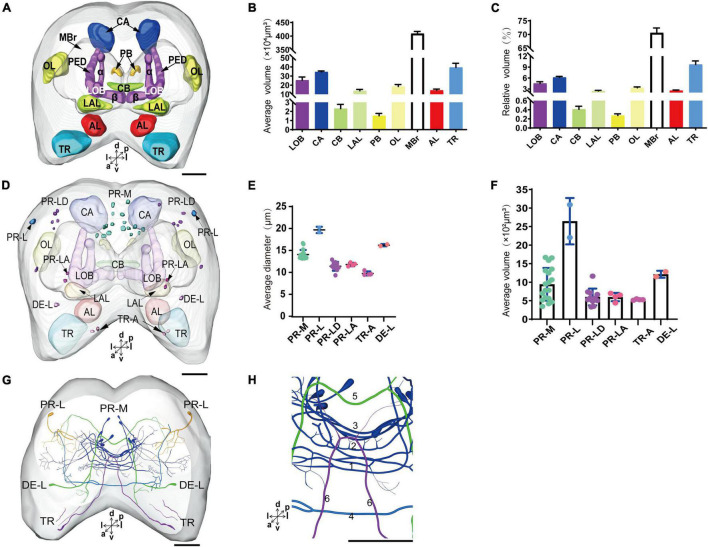
The average and relative volume of the neuropils, the average diameter and volume of cell bodies, and the distribution of the serotonergic neuronal processes and cell bodies in the brain. **(A)** Three-dimensional reconstructions of the neuropils in the brain in frontal view. **(B,C)** The average and relative volume of the neuropils. **(D)** Reconstructions of cell bodies in the brain. **(E,F)** The average diameter and volume of cell bodies in the brain. **(G)** Reconstructed skeleton trees of the thick neuronal processes showing their projection patterns in anterior view. **(H)** Reconstructed skeleton trees of commissures 1–6 in anterior view. PR-M, PR-L, PR-LD, PR-LA, DE-L, and TR-A are cell clusters. α, alpha lobe; AL, antennal lobe; β, belta lobe; CA, calyx; CB, central body; LAL, lateral accessory lobe; LOB, mushroom body lobes; OL, optic lobe; PB, protocerebral bridge; TR, tritocerebrum. Directions: a, anterior; d, dorsal; l, lateral; m, medial; p, posterior; v, ventral. Scale bar, 100 μm.

The immunoreactivity of anti-SYNORF1 revealed the synapsin-enriched neuropil and that of antiserotonin revealed cell bodies and cell fibers of serotonergic neurons in the brain (Figures 1A1–A5, merged; B1-B5, anti-SYNORF1; C1-C5, antiserotonin). The brain of larval *S. frugiperda* contained three main neuromeres: the protocerebrum (PR), the deutocerebrum (DE), and the tritocerebrum (TR). Based upon the intensity of immunoreactivity, several prominent neuropils in the PR could be identified (Figures 1B1–B5). Three-dimensional reconstructions of the neuropils were also created and then their volumes and relative volumes to those of the whole brain were measured (Figure 2). The alpha lobe (α) was located anteriorly and pointed vertically to the dorsal surface and the beta lobe (β) was located medially and pointed horizontally to the middle line of the PR (Figures 1B1,B2, 2A). The pedunculus (PED) lay in the middle of each hemisphere, forming the lobe (LOB) of mushroom body together with the α and β lobes (Figures 1B3,B4, 2A). Volume of LOB is 25.44 × 10^4^ μm^3^, about 4.55% of the whole-brain neuropil (Figures 2B,C). The calyx (CA) was located posteriorly in the PR (Figures 1B3,B4, 2A). Volume of CA is 34.54 × 10^4^ μm^3^, about 6.17% of the whole-brain neuropil (Figures 2B,C). The central body (CB), an unpaired neuropil, was located horizontally in the center of the PR, crossed the midline, and linked both the hemispheres (Figures 1B3, 2A). Volume of CB is 2.34 × 10^4^ μm^3^, about 0.41% of the whole-brain neuropil (Figures 2B,C). The lateral accessory lobe (LAL) was also visible, located laterally to the mushroom body lobes (Figures 1B3, 2A). Volume of LAL is 13.84 × 10^4^ μm^3^, about 2.47% of the whole-brain neuropil (Figures 2B,C). The protocerebral bridge (PB) was located posteriorly, on either side of middle line of the PR (Figures 1B4, 2A). Volume of PB is 1.53 × 10^4^ μm^3^, about 0.27% of the whole-brain neuropil (Figures 2B,C). The optical lobe (OL) was located on most lateral side of the PR (Figures 1B5, 2A). Volume of OL is 18.60 × 10^4^ μm^3^, about 3.33% of the whole-brain neuropil (Figures 2B,C). In addition to these prominent neuropils mentioned above, the PR also contained a large neuropil, referred as midbrain (MBr), which has homogeneous intensity of immunoreactivity without obvious boundaries. Volume of MBr is 397.28 × 10^4^ μm^3^, about 70.57% of the whole-brain neuropil (Figures 2B,C). The antennal lobe (AL), a spherical structure of the DE, was located most anteriorly of the brain (Figures 1B1, 2A). The volume of AL is 14.38 × 10^4^ μm^3^, about 2.57% of the whole-brain neuropil (Figures 2B,C). The TR was located most ventral of the brain, at the root of circumesophageal connective, which linked the brain and the gnathal ganglion (Figures 1B1–B3, 2A). Volume of the TR is 54.74 × 10^4^ μm^3^, about 9.72% of the whole-brain neuropil (Figures 2B,C).

### Serotonergic Neurons in the Brain of Larval *Spodoptera frugiperda*

**FIGURE 3:**
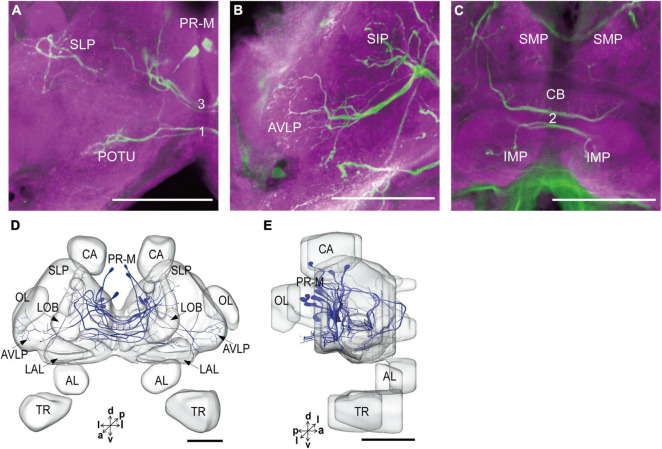
The projection area of PR-M neurons. **(A–C)** The confocal stack images of PR-M neurons. **(D)** The anterior view of three-dimensional reconstructions of PR-M neurons. **(E)** The lateral view of three-dimensional reconstructions of PR-M neurons. AVLP, anterior ventrolateral protocerebrum; CB, central body; IMP, inferior medial protocerebrum; SLP, superior lateral protocerebrum; SMP, superior medial protocerebrum; POTU, posterior optic tubercle.PR-M is cell cluster. Directions: a, anterior; d, dorsal; l, lateral; p, posterior; v, ventral. Scale bar, 100 μm.

The serotonergic neurons revealed by the immunoreactivity to antiserotonin serum had their cell bodies in the cell body layer and neural fibers projected in wide regions of the neuropils and crossed the midline forming six commissures linking both the hemispheres (Figures 1C1–C5). All the identified cell bodies were counted and their diameters and volumes were measured (Figures 2D–F). There are about 40 serotonergic neurons in the brain (Table 1). The cell body cluster of PR-M was located in the medial region of the posterior PR and contained 18 cell bodies, nine in each hemisphere. Diameters of these cell bodies are in the range of 12.19–16.66 μm and volumes are in the range of 407–1,531 μm^3^. Serotonergic neurons of PR-M were bilateral and extended widespread projection to the neuropils of the both hemisphere protocerebra via the commissures 1–3 (Figures 2G,H, 3). Innervation regions of their terminals include posterior optic tubercle (POTU), anterior ventrolateral protocerebrum (AVLP), superior intermediate protocerebrum (SIP), superior medial protocerebrum (SMP), inferior medial protocerebrum (IMP), and CB (Figure 3). However, the neuropils of PB, CA, PED, α and β lobes, and lateral horn lack serotonergic neuron processes.

**TABLE 1:** Number, location, and innervation areas of serotonin-immunoreactive neurons in the brain and the gnathal ganglion of *Spodoptera frugiperda* larvae.

Brain/GNG	Cell body cluster	Number of neurons (n)	Location of cell body	Innervation areas
Brain	PR-M	16–18(4)	Medial region of the posterior protocerebrum	Posterior optic tubercle, anterior ventrolateral protocerebrum, superior intermediate protocerebrum, superior medial protocerebrum, inferior medial protocerebrum, and central body
	PR-L	2(4)	Lateral region of the lateral protocerebrum	Ipsilateral regions of optic lobe, posterior lateral protocerebrum, and superior lateral protocerebrum
	PR-LD	10–12(4)	Dorsally to the lateral protocerebrum	Not resolved
	PR-LA	4(4)	Anteriorly to the lateral protocerebrum	Not resolved
	TR-A	4(4)	Anteromedial tritocerebrum	Contralateral superior medial protocerebrum
GNG	GNG-AD	7(5)	Medial region of anteriodorsal gnathal ganglion	Anterior mandibular neuromere and tritocerebrum
	GNG-AV	2(5)	Anterior region of ventral gnathal ganglion	Not resolved
	GNG-M	3(5)	Median area of ventral gnathal ganglion	Not resolved
	GNG-L1	4(5)	Lateral cell body layer to the mandibular neuromere	Mandibular neuromere and tritocerebrum
	GNG-L2	4–5(5)	Lateral cell body layer to the maxillary neuromere	Maxillary neuromere and tritocerebrum
	GNG-L3	4(5)	Lateral cell body layer to the labial neuromere	Labial neuromere and tritocerebrum

**FIGURE 4:**
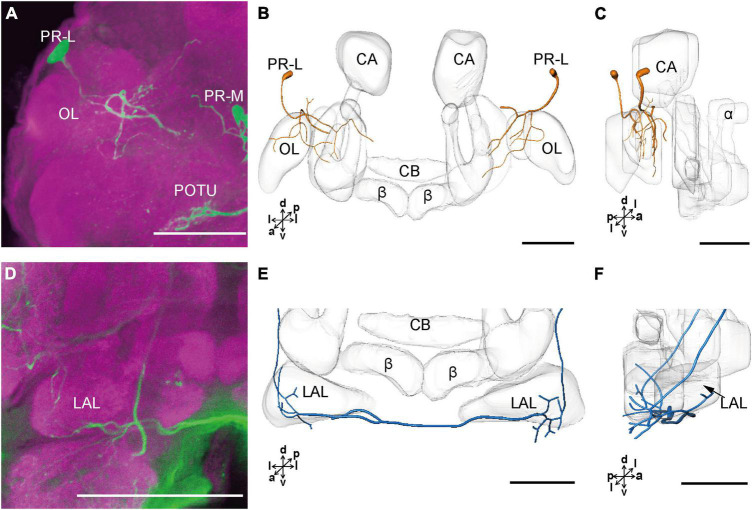
The projection area of PR-L and LAL neurons. **(A)** The confocal stack images of PR-L neurons. **(B,C)** The anterior and lateral view of the three-dimensional reconstructions of the PR-L neurons. **(D)** The confocal stack images of LAL neuron. **(E,F)** The three-dimensional reconstructions of the LAL neurons in the anterior and lateral views. α, alpha lobe; β, belta lobe; CA, calyx; CB, central body; LAL, lateral accessory lobe; OL, optic lobe; POTU, posterior optic tubercle; PR-M and PR-L are cell clusters. Directions: a, anterior; d, dorsal; l, lateral; p, posterior; v, ventral. Scale bars, 100 μm.

The cell body of PR-L was located in the lateral region of the lateral PR, only one in each hemisphere (Figure 2D). Diameters of these cell bodies are in the range of 17.31–20.01 μm and volumes are in the range of 2,199–3,089 μm^3^ (Figures 2E,F). Its axon projected to ipsilateral regions of optic lobe, posterior lateral protocerebrum (PLP), and superior lateral protocerebrum (SLP) (Figures 4A–C and Table 1). There were 10 labeled cell bodies in the cluster of PR-LD, located dorsally to the lateral PR (Figure 2D). Diameters of these cell bodies are in the range of 9.15–13.81 μm and volumes are in the range of 431–1,162 μm^3^ (Figures 2E,F). There were 4 labeled cell bodies in the cluster of PR-LA, located anteriorly to the lateral PR (Figure 2D and Table 1). Diameters of these cell bodies are in the range of 9.46–10.46 μm and volumes are in the range of 512–550 μm^3^ (Figures 2E,F). No visible processes were observed from PR-LD and PR-LA. Two LALs were also innervated by serotonin neurons and linked by commissure 4 (Figures 2G,H, 4D–F); however, the cell bodies for these serotonergic neurons were unable to be traced.

**FIGURE 5:**
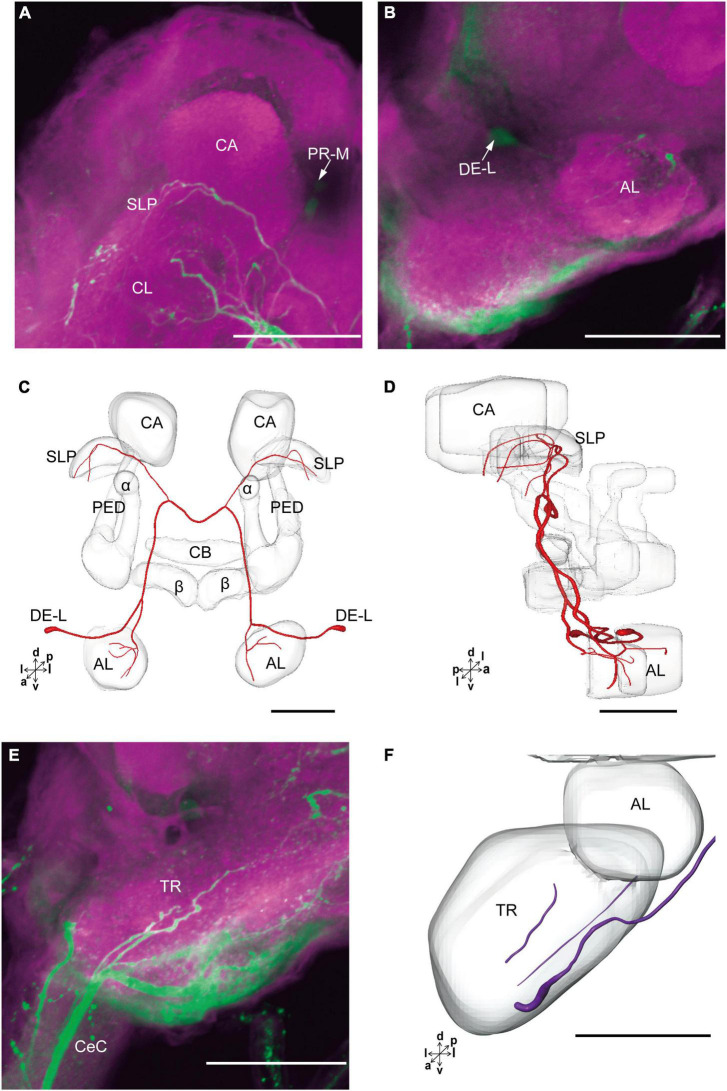
The projection area of DE-L and TR neurons. **(A,B)** The confocal stack images of DE-L neurons. **(C,D)** The anterior and lateral views of the three-dimensional reconstructions of the DE-L neurons. **(E)** The confocal stack images of TR neuron. **(F)** The three-dimensional reconstructions of the TR neurons in anterior view. α, alpha lobe; AL, antennal lobe; β, belta lobe; CA, calyx; CB, central body; CeC, circumoesphageal connective; PED, pedunculus; SLP, superior lateral protocerebrum; PR-M and DE-L are cell clusters. Directions: a, anterior; d, dorsal; l, lateral; p, posterior; v, ventral. Scale bars, 100 μm.

The cluster of DE-L contained only one cell body located laterally to the AL on each hemisphere (Figure 2D and Table 1). Diameters of these cell bodies are in the range of 15.37–16.91 μm and volumes are in the range of 1,213–1,728 μm^3^ (Figures 2E,F). The neurites of DE-L ran dorsoposteriorly into the ipsilateral PR via medial antennal lobe tract, crossed the midline via commissure 5, and then projected via contralateral medial antennal tract to the contralateral AL. In addition, this neuron also gave rise to arborizations in both the hemispheres of SLP (Figures 2G,H, 5A–D and Table 1).

There were four cell bodies in TR-A of both the hemispheres (Figure 2D). Diameters of these cell bodies are in the range of 10.92–12.57 μm and volumes are in the range of 440–715 μm^3^ (Figures 2E,F). No visible processes, however, were observed from TR-A. The TR, indeed, contained the processes of serotonergic neurons, which were from the neurons in the gnathal ganglion via the circumesophageal connective (Figure 5E). In addition, a single serotonergic neuron passing the TR, projected upward along the medial side, and then crossed the midline giving off arborizations in the contralateral superior medial PR (Figures 2G,H, 5E,F and Table 1).

### Anatomy and Serotonergic Neurons of the Gnathal Ganglion of Larval *Spodoptera frugiperda*

**FIGURE 6:**
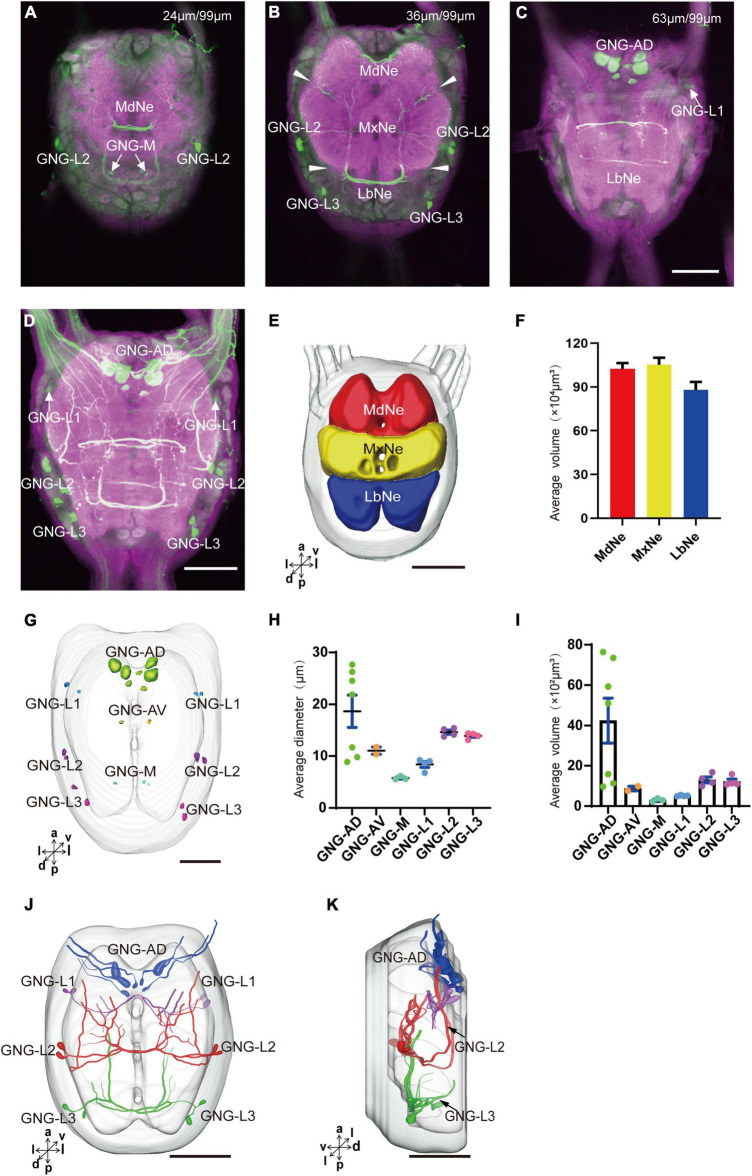
Neuromeres, cell bodies, and processes of the serotonergic neuron in the gnathal ganglion. **(A–C)** Confocal images showing the distribution of the serotonergic neuron processes and cell bodies in the gnathal ganglion. **(D)** The confocal stack images of gnathal ganglion. **(E)** Three-dimensional reconstructions of main neuromeres in gnathal ganglion. **(F)** The average volume of main neuromeres. **(G)** The location of cell bodies in gnathal ganglion. **(H,I)** The average diameter and volume of cell bodies in gnathal ganglion. **(J,K)** Three-dimensional reconstructions of the serotonergic neurons in the cluster of gnathal ganglion in anterior and lateral view. GNG-AD, GNG-AV, GNG-M, GNG-L1, GNG-L2, and GNG-L3 are cell clusters. MdNe, mandibular neuromere, MxNe, maxillary neuromere, LbNe, labial neuromere. Directions: a, anterior; d, dorsal; l, lateral; m, medial; p, posterior; v, ventral. Scale bars, 100 μm.

The gnathal ganglion was composed of three neuromeres: the mandibular neuromere (MdNe), the maxillary neuromere (MxNe), and the labial neuromere (LbNe) (Figures 6A–E). The average volume of three neuromeres were about 87.92–105.17 × 104 μm^3^ (Figure 6F). There were about 24 cell bodies of serotonergic neurons distributed in several clusters of GNG-AD, GNG-AV, GNG-M, and GNG-L (Figures 6A–D,G).

GNG-AD contained seven cell bodies, situated in the medial region of anterodorsal gnathal ganglion. Diameters of these cell bodies are in the range of 8.87–26.27 μm and volumes are in the range of 965–7,847 μm^3^ (Figures 6H,I). These neurons sent their arborization into the TR. GNG-AV contained two cell bodies, located in the anterior region of ventral gnathal ganglion. Diameters of these cell bodies are in the range of 9.34–10.79 μm and volumes are in the range of 758–1,038 μm^3^ (Figures 6H,I). GNG-M contained three cell bodies, located in the median area of ventral gnathal ganglion. Diameters of these cell bodies are in the range of 3.53–4.17 μm and volumes are in the range of 305–381 μm^3^ (Figures 6H,I). No visible processes were observed from neurons of GNG-AV and GNG-AD.

Three clusters, GNG-L1, GNG-L2, and GNG-L3, were located in the lateral cell body layer to the MdNe, the MxNe, and the LbNe, respectively. Each cluster contained four cell bodies with two on each hemisphere. Diameters of these cell bodies are in the range of 8.56–14.41 μm and volumes are in the range of 607–1,799 μm^3^ (Figures 6H,I). These neurons sent their thick axons to the contralateral hemisphere via the commissure, gave off many fine arborizations, and then projected upward to the TR through the circumesophageal connective (Figures 6J,K and Table 1).

## Discussion

Neuropil structures and serotonergic neurons of the brain and the gnathal ganglion of larval *S. frugiperda* were identified based upon the immuoreactivities with antisynapsin and antiserotonin serum. The gnathal ganglion of larval *S. frugiperda* is a neuropil fused with the MdNe, the MxNe, and the LbNe and the brain fused with the PR, the DE, and the TR. Within the PR, the neuropils of OL, LOB, CA, CB, PB, and LAL are prominent and easily identified. The other neuropils of the PR including the lateral PR and the superior PR account for 70.57% of the brain, showing no obvious boundaries. The prominent neuropil of the DE is AL. Structure and spatial arrangement of the brain neuropils are similar to that of studied lepidoptera species, for instance, the monarch butterfly *Danaus plexippus*, the sphinx moth *Manduca sexta* (*M. sexta*), the cotton bollworm *Helicoverpa armigera* (*H. armigera*), and the tea geometrid *Ectropis obliqua* (*E. obliqua*) (Nordlander and Edwards, 1968; Huetteroth et al., 2010; Tang et al., 2014; Xie et al., 2016). The volumes and the relative volume of the prominent neuropils are also similar to those of *H. armigera* and *E. obliqua* (Tang et al., 2014; Xie et al., 2016).

### Number and Size of Serotonergic Neurons in the Brain and the Gnathal Ganglion

There are about 40 serotonergic neurons in the brain and about 24 serotonergic neurons in the gnathal ganglion of *S. frugiperda* larvae. The similar numbers were also reported in other larvae species, moths *M. sexta* (Granger et al., 1989; Griss, 1989), *H. armigera* (Tang et al., 2019), flies *Drosophila melanogaster* (*D. melanogaster*) (Vallés and White, 1988; Huser et al., 2012), *Calliphora erythrocephala* and *Sarcophaga bullata* (Nässel and Cantera, 1985), and the beetle *Tenebrio molitor* (Breidbach, 1990). In adults of *M. sexta* and *D. melanogaster*, the number of serotonergic neurons in the gnathal ganglion is also about 20 (Vallés and White, 1988; Homberg and Hildebrand, 1989a; Sitaraman et al., 2008). Similarly, in the central brain of adults of these species, e.g., brain neuropils excluding the optic lobe, the number of serotonergic neurons is about 40 in *M. sexta*, *D. melanogaster*, the honeybee *Apis mellifera* (*A. mellifera*), the wasp *Trichogramma evanescens* (*T. evanescens*), and the blood-feeding bug *Triatoma infestans* (Schürmann and Klemm, 1984; Lange et al., 1988; Vallés and White, 1988; Homberg and Hildebrand, 1989a; Sitaraman et al., 2008; van der Woude and Smid, 2017). No cell bodies of serotonergic neurons were found in the optic lobe of *S. frugiperda*. The similar results were also observed in *H. armigera* (Tang et al., 2019). However, in adults, there are about 600 serotonergic neurons located in the optic lobe of *M. sexta*, 80 in armyworm *Mythimna separata*, 100 in butterfly *Mimathyma schrenckii* (*M. schrenckii*), 40 in *D. melanogaster*, 40–60 in *A. mellifera*, and 120 in mantis *Tenodera sinensis* (Schürmann and Klemm, 1984; Vallés and White, 1988; Homberg and Hildebrand, 1989b; Leitinger et al., 1999; Niu et al., 2004; Sitaraman et al., 2008; Guo et al., 2018). The differentiation of serotonergic neurons in the optic lobe is dependent on the development of optic lobe neuropils during the metamorphosis (Nässel et al., 1987).

Cell body sizes of serotonergic neurons of *S. frugiperda* larvae were also measured. Cell bodies in diameters are about 9–20 μm in the brain and 3.5–26 μm in the gnathal ganglion. In *T. evanescens*, the diameters of cell body sizes of serotonergic neurons are about 2 μm, while in *A. mellifera*, the diameters of cell body sizes of serotonergic neurons are 8–30 μm (Schürmann and Klemm, 1984; van der Woude and Smid, 2017). Therefore, the size of serotonergic neurons in different species may be related to insect body sizes (van der Woude and Smid, 2017). Within a species, however, why the size of serotonergic neurons differ from different clusters is not clear. The size of cell body may be related to the size of arborization. For example, the cell bodies of PR-M, PR-L, and DE-L in *S. frugiperda* larvae are larger and their arborizations are spread wider. The size of cell body may also be related to the function of neurosecretion. Four serotonergic neurons of GNG-AD located in the medial gnathal ganglion are very large. Similar results were also found in locust *Schistocerca gregaria*, cockroach *Periplaneta americana*, larval *H. armigera*, and larval and adult *M. sexta* (Bishop and O’Shea, 1983; Tyrer et al., 1984; Griss, 1989; Homberg and Hildebrand, 1989a; Tang et al., 2019). Intracellular recordings from such neurons of *M. sexta* larvae revealed overshooting soma spikes of large amplitude and long duration, which suggest that these neurons are neurosecretory cells (Griss, 1989).

### Innervation Patterns of Serotonergic Neurons in the Brain

In the PR, the cell bodies of serotonergic neurons of larval *S. frugiperda* are distributed mainly in four clusters: PR-M, PR-L, PR-LD, and PR-LA. The processes of PR-LD and PR-LA are invisible; probably, they are not developed yet in the present stage. In contrast, PR-M has processes projecting to wide region in the PR, including the CB, bilateral areas of superior intermediate protocerebra, superior and inferior medial protocerebra, anterior ventrolateral protocerebra, and POTU. The arborizations in these areas are quite dense. The neurons in the cluster of PR-L have processes projecting mainly to the ipsilateral posterior and superior lateral protocerebra. A few processes of these neurons project to the inner part of the ipsilateral optic lobe. Genetic manipulations demonstrated that a subset of serotonergic neurons in the anterior, medial, and lateral PR evoked hunger (Albin et al., 2015) and several serotonergic neurons in the inferior PR and the lateral PR inhibited the attraction of ethanol in adult *D. melanogaster* (Xu et al., 2016). Many processes were also found in both the LALs of larval *S. frugiperda*; however, their cell bodies were unable to be traced. The serotonergic processes in the LAL of *H. armigera* originated from the cells in the cluster of PR-A, which located in anterior region of the PR (Tang et al., 2019). The patterns of serotonergic neuron arborizations of PR-M and PR-L are similar between *S. frugiperda* and *H. armigera*. The neuropils of the PR of larval *S. frugiperda*, including PB, CA, PED, α and β lobes, and lateral horn, lack serotonergic neuron processes. Similar findings have been reported in larvae of *H. armigera*, *M. sexta*, and *D. melanogaster* (Granger et al., 1989; Huser et al., 2012; Tang et al., 2019). In contrast, the mushroom bodies of adult *M. sexta* and *D. melanogaster* contain fine serotonergic neuron processes (Homberg and Hildebrand, 1989a; Sitaraman et al., 2008). These results suggest that some serotonergic neurons in the mushroom body are remodeled during the metamorphosis from the larva to the adult. The mushroom bodies of insects are related to learning and memory activities and serotonin have been demonstrated to be involved in olfaction and place learning and memory in *D. melanogaster* (Sitaraman et al., 2008, 2012). The lack of serotonergic neuron in the larval mushroom body, however, does not suggest that serotonin plays no role in learning and memory at larval stage. Recently, a serotonin receptor, 5-HT7, was found expressing in the mushroom body of *Drosophila* larvae, which was shown to mediate the associative olfactory appetitive learning and memory (Huser et al., 2017; Ganguly et al., 2020). Whether serotonin mediates the associative learning and memory for *S. frugiperda* in the same manner could be investigated by using molecular methods in future study.

A pair of deutocerebral serotonergic neurons DE-L of larval *S. frugiperda* has arborizations in the contralateral AL and bilateral superior lateral protocerebra, which was similar to the reports in the larvae of *M. sexta* and *H. armigera* (Kent et al., 1987; Tang et al., 2019). In adult of *M. sexta*, the branching pattern persists and expands in the AL with the development of glomeruli (Kent et al., 1987). The similar arborization patterns between larvae and adult may indicated DE-L neurons that play the same function in both the different life stages. Electrophysiological recordings demonstrated that the deutocerebral serotonergic neuron showing responses to odorants and mechanical stimuli in adult moths *Bombyx mori* (*B. mori*) and *Helicoverpa assulta* (Hill et al., 2002; Zhao and Berg, 2009). In *D. melanogaster*, these two deutocerebral serotonergic neurons could counteract the inhibition of the ethanol attraction from the serotonergic neurons of the PR (Xu et al., 2016). In *B. mori*, dye-filled DE-L neuron also gave off some arborizations in the lateral accessory lobe, but such innervation pattern was not found in larval *S. frugiperda* or other studied lepidopteran species (Dacks et al., 2006; Zhao and Berg, 2009; Tang et al., 2019).

The cells in the cluster of TR-A in the TR of larval *S. frugiperda* were also similar to that of *H. armigera* and other species (Nässel, 1988; Granger et al., 1989; Wegerhoff, 1999; Tang et al., 2019). The cell bodies were weakly stained with antiserotonin serum and their neuronal processes were not detected. Throughout the TR, however, serotonergic neuron processes are abundant and they may originate from the frontal ganglion and the gnathal ganglion. Two neurons, which linked the TR, form a commissure in the frontal of the medial PR and give off some arborizations in the SMP. The TR is the stomatogastric center. The findings of the serotonergic neuron network between the TR, the PR, and the gnathal ganglion could facilitate us to explore the roles of serotonin in feeding, for instance, food detection, food intake, and nutrient choice, and help in finding a potential approach to modulate the feeding behavior of the gluttonous pest and reduce its damage.

### Serotonergic Neurons in the Gnathal Ganglion

The immunoreactivity to antiserotonin serum in the cell cluster of GNG-AV and GNG-M of larval *S. frugiperda* was weak and their processes were unable to be traced. The cluster of GNG-AD gave off processes in the most anterior of the gnathal ganglion and projected to the TR. All the three neuromeres of the gnathal ganglion of larval *S. frugiperda* contain widespread processes of serotonergic neurons originated from the cells in the cluster GNG-L. The thick processes from the cell clusters on both the sides form a horseshoe pattern, cross the midline via a commissure, and project anteriorly to the contralateral TR. Such neurons and their branching patterns show high conservation across insect taxa, which were also found in larvae of *H. armigera*, *M. sexta*, *Tenebrio molitor* (*T. molitor*) and the flies *D. melanogaster*, *Conistra erythrocephala* (*C. erythrocephala*), and *Sarcophaga bullata* (*S. bullata*) (Nässel and Cantera, 1985; Griss, 1989; Breidbach, 1990; Huser et al., 2012; Tang et al., 2019).

In adults of insect species, serotonergic neurons and their processes in the gnathal ganglion were also found in the similar patterns (Bishop and O’Shea, 1983; Tyrer et al., 1984; Rehder et al., 1987; Vallés and White, 1988; Griss, 1989; Homberg and Hildebrand, 1989a; Breidbach, 1990). Previous studies demonstrated that the serotonergic neurons in the lateral side of the gnathal ganglion facilitated the food ingestion of *D. melanogaster* larvae (Schoofs et al., 2018). In addition to the internal regulation, the serotonergic neurons of the gnathal ganglion were also involved in mediating taste detection (Yao and Scott, 2021). One class of serotonergic neurons in the gnathal ganglion responds to gustatory detection of sugars and the other class to gustatory detection of bitter compounds (Yao and Scott, 2021). As in other species, larval *S. frugiperda* possesses taste sensilla on the maxilla, responding to the stimuli sugar and bitter substances to regulate the feeding preference (Hou et al., 2020). How the serotonergic neurons in the gnathal ganglion of larval *S. frugiperda* regulate the feeding preference would be an interesting issue in the future study.

## Data Availability Statement

The raw data supporting the conclusions of this article will be made available by the authors, without undue reservation.

## Author Contributions

Q-BT, W-BC, and X-CZ: study concept and design. J-JZ, L-LS, Y-NW, G-YX, and W-BC: acquisition of data. J-JZ, L-LS, G-YX, W-BC, and X-CZ: analysis and interpretation of data. W-BC and X-CZ: drafting of the manuscript. Q-BT, W-BC, X-CZ, and S-HA: final manuscript. Q-BT and W-BC: obtain funding. All authors contributed to the article and approved the submitted version.

## Conflict of Interest

The authors declare that the research was conducted in the absence of any commercial or financial relationships that could be construed as a potential conflict of interest.

## Publisher’s Note

All claims expressed in this article are solely those of the authors and do not necessarily represent those of their affiliated organizations, or those of the publisher, the editors and the reviewers. Any product that may be evaluated in this article, or claim that may be made by its manufacturer, is not guaranteed or endorsed by the publisher.
